# Multi-Task Learning of Scanning Electron Microscopy and Synthetic Thermal Tomography Images for Detection of Defects in Additively Manufactured Metals

**DOI:** 10.3390/s23208462

**Published:** 2023-10-14

**Authors:** Sarah Scott, Wei-Ying Chen, Alexander Heifetz

**Affiliations:** 1 Nuclear Science and Engineering Division, Argonne National Laboratory, Lemont, IL 60439, USA; sarah.scott@duke.edu (S.S.); wychen@anl.gov (W.-Y.C.); 2 Department of Civil and Environmental Engineering, Duke University, Durham, NC 27708, USA

**Keywords:** multi-task learning, deep learning, computer vision, thermal tomography, scanning electron microscopy, additive manufacturing of metals, regression–classification, semantic segmentation

## Abstract

One of the key challenges in laser powder bed fusion (LPBF) additive manufacturing of metals is the appearance of microscopic pores in 3D-printed metallic structures. Quality control in LPBF can be accomplished with non-destructive imaging of the actual 3D-printed structures. Thermal tomography (TT) is a promising non-contact, non-destructive imaging method, which allows for the visualization of subsurface defects in arbitrary-sized metallic structures. However, because imaging is based on heat diffusion, TT images suffer from blurring, which increases with depth. We have been investigating the enhancement of TT imaging capability using machine learning. In this work, we introduce a novel multi-task learning (MTL) approach, which simultaneously performs the classification of synthetic TT images, and segmentation of experimental scanning electron microscopy (SEM) images. Synthetic TT images are obtained from computer simulations of metallic structures with subsurface elliptical-shaped defects, while experimental SEM images are obtained from imaging of LPBF-printed stainless-steel coupons. MTL network is implemented as a shared U-net encoder between the classification and the segmentation tasks. Results of this study show that the MTL network performs better in both the classification of synthetic TT images and the segmentation of SEM images tasks, as compared to the conventional approach when the individual tasks are performed independently of each other.

## 1. Introduction

Laser powder bed fusion (LPBF) is an emerging metal additive manufacturing (AM) method for the fabrication of custom, complex shape structures from high-strength alloys for applications in harsh environments [1]. The LPBF process involves developing a 3D drawing of a structure using either computer-aided design (CAD) or 3D scans of an object to be replicated. The CAD file is then uploaded on a computer controlling the 3D metal printer. The LPBF printer creates the structure by sequentially depositing layers of microscopic grains and using high-power laser beams to selectively melt the powder grains in each layer. Because of its features, LPBF is a promising enabling technology for nuclear energy sustainability through the cost-efficient fabrication of replacement metallic components for aging commercial light water reactors [2,3]. One of the main challenges to the acceptance of LPBF for nuclear manufacturing is the appearance of microscopic porosity defects in 3D-printed metal structures. Such pores are an artifact of the metal AM process involving rapid melting and solidification without well-defined boundary conditions [4]. Depending on the size, shape, and orientation relative to structure surfaces, porosity defects could be initiating sites for material crack formation, and thus lead to premature structural failure [5,6,7].

The approaches to quality control of AM structures involve in situ monitoring of structure layers during printing [8,9,10,11], and ex situ evaluation of the final printed structures [12,13,14,15]. Regardless of in situ monitoring results, the detection and classification of material defects in the final structure prior to deployment in a nuclear reactor is necessary because of the stringent safety requirements of nuclear energy. There exist several approaches to ex situ quality control of printed metallic LPBF structures based on destructive analysis [16,17,18] and non-destructive evaluation (NDE) [19,20]. The goal of this paper is to introduce a new machine learning algorithm that simultaneously performs analysis of the images obtained from ex situ destructive and non-destructive sensing modalities. The joint learning process results in a better performance compared to that for learning of individual tasks separately.

Destructive testing typically involves examining coupons to obtain statistical representation of the quality of the print process. Sectioning or chemically etching the specimen, and imaging section surfaces with a scanning electron microscope (SEM) offers an efficient approach to rapid imaging, with minimal specimen preparation time, of relatively large sections of specimens with a spatial resolution as low as 10 nm/pixel [16,17]. Random sections of metallic specimens contain cavities, which correspond to cross-section cuts through irregular-shaped microscopic pores. Cavity characteristics (size and shape) obtained from the SEM images thus provide information about the quality of metal printing. Analysis of SEM images can be performed with semantic segmentation, which performs pixel-wise classification, outputting segmentation maps in which each pixel in the image belongs to a pre-defined class.

Because of the low reproducibility of AM metals, as compared to conventional manufacturing, the imaging of actual structures intended for service in a nuclear reactor is needed for safety verification. In principle, NDE with high imaging resolution (on the order of microns) can be performed with X-ray computed tomography (XCT) [13,14]. However, XCT requires specimens with body-of-revolution symmetry (e.g., spheres and cylinders), and high resolution is limited to small metallic coupons with dimensions on the order of millimeters. Ultrasonic testing (UT) is an NDE method scalable to arbitrary structure sizes and shapes, but requires direct contact with the structure surface [19,20]. This limits the applicability of UT because LPBF-printed metals have rough surfaces due to the specifics of the manufacturing process. Pulsed infrared thermography (PIT) or the active thermography NDE method use non-contact sensors for the imaging of subsurface defects in optically opaque materials [21,22,23,24,25,26,27,28]. PIT uses a flash lamp to deposit a thermal pulse on a material surface. As heat diffuses into the material bulk, a fast frame infrared camera records the surface temperature transients, via capturing the emission of blackbody radiation from the solid [12]. The presence of internal pores in the material is revealed via the appearance of temperature “hot spots” on the material surface above the flaw due to slower heat decay caused by air-filled pore with higher thermal resistance. Typically, mid-wave IR cameras with a 3–5 µm spectral band are used for imaging, which, in principle, allows for a diffraction-limited resolution of 5 µm per pixel.

The resulting data cube in PIT consists of sequential frames of temperature distribution in mixed spatial and temporal coordinates (x,y,t). The thermal tomography (TT) computational method uses PIT data to obtain the reconstruction of the thermal effusivity of spatial-only coordinates (x,y,z). Spatial depth reconstruction allows for the visualization of material defects [29]. The interpretation of TT images is not trivial, in part because imaging is based on heat diffusion, and thus images suffer from blurring, which increases with depth. Machine learning algorithms including K-Means Singular Value Decomposition and sparse dictionary learning [30] have been developed to compensate for the blurriness of TT reconstructions. To classify material defects (size and orientation) from TT images, we have recently developed a machine learning approach based on a convolutional neural network (CNN) [31]. A CNN is an image analysis algorithm, where input data is paired with corresponding labels in a supervised manner. Consisting of multiple hierarchical convolutional layers, the CNN networks efficiently identify distinctive patterns and features within images by performing classification and regression tasks. In classification tasks, features extracted from images are used to assign a predefined class value to the input image, and in regression tasks, the features extracted from CNNs are used to predict appropriate continuous values. In prior studies, CNN was shown to be an efficient method for analysis of images for in situ monitoring of AM [8,9,10,11,12], and NDE of AM [23,24,25,26,27,28].

This paper presents a novel approach to improving the accuracy of parameter prediction of TT images by using joint multi-task learning (MTL) on two disjoint databases of TT and SEM images of material defects [32]. In the MTL approach, the same network is employed to perform multiple tasks simultaneously, taking inputs from separate yet related tasks, to make the predictive ability of the network more robust. The presence of multiple tasks enables the network to leverage enhanced extracted features, leading to improved multitasking abilities. This is analogous to the increase in human cognitive performance resulting from learning to play a new instrument [33,34], or the increase in the human academic performance with increased gross motor skills [35]. Recent work on MTL has been primarily in the medical applications domain, such as development of MTL models for the segmentation of ultrasound and magnetic resonance images (MRI) of tumors [36,37,38]. There has been a limited number of studies of MTL for AM applications, with examples that include multi-task Gaussian processes for modeling shape deviations and surface modeling of manufactured parts [39,40,41].

Advantages of the MTL include relative simplicity of the network architecture, where a shared encoder for the classification of TT images and segmentation of SEM images tasks. In the MTL network in this paper, a CNN-based regression is performed on a dataset of synthetic TT images of elliptical defects in stainless steel, developed in the prior study [31]. In practice, experimental measurement datasets typically have a moderate size with limited variance. However, extensive datasets encompassing large variance in the data are needed for machine learning (ML) model training to achieve sufficient accuracy in predictions. Recent research approaches to address this challenge have investigated the use of synthetic (simulated) and augmented (modulated) NDE data for ML model development [42,43,44,45,46,47]. In line with this approach, future work with experimental TT data will involve the use of synthetic and augmented data for ML model development. Therefore, the present study with synthetic TT images provides valuable benchmarking results of MTL algorithm performance.

MTL network segmentation is performed on a set of SEM image sections of LPBF-printed stainless steel 316 specimens. Current state of the art in segmentation includes fully convolutional neural networks (FCNNs) [48]. However, FCNNs typically require a large volume of training data. Because the sectioning of specimens represents the random sampling of sparse internal pores in the meta, the fraction of SEM images containing material defects is relatively small. Because of the limited training data, we perform segmentation of SEM images using the traditional U-Net architecture better suited for “from-scratch” training on smaller datasets [49]. By jointly training the MTL network on the regression of TT images and segmentation of SEM images tasks, we exploit shared knowledge and feature representations, enabling the MTL network to acquire a deeper understanding of image processing. To the best of our knowledge, the work in this paper is the first demonstration of using MTL utilizing U-Net for simultaneous information processing from disjoint datasets of images of material defects. The results of this study demonstrate the efficiency of the MTL approach, which results in improvements in image processing performance, as compared to performance of the individual regression and segmentation tasks separately.

## 2. Multi-Task Learning Network for Classification and Segmentation of Images

### 2.1. Datasets of Synthetic Thermal Tomography and Scanning Electron Microscopy Images

**Figure 1:**
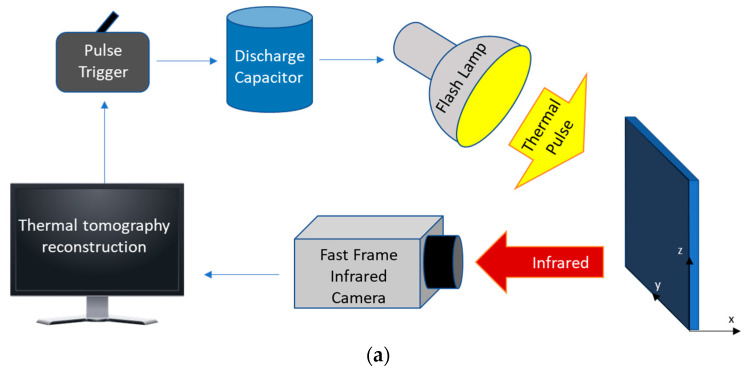

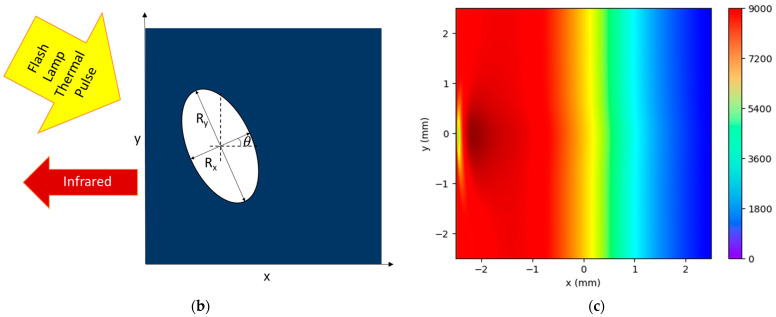
(**a**) Schematics of PIT system data acquisition. (**b**) Diagram of an elliptical material defect considered in this study. (**c**) Pseudo color image visualization of thermal effusivity reconstruction *e*(x,y) [J∙m^−3/2^∙(mK∙s)^−1/2^] for an SS316 plate containing an elliptical defect with parameters *R*_x_ = 60 µm, *R*_y_ = 310 µm, and *θ =* 15°.

The dataset for the regression–classification task consists of synthetic TT images of 2D stainless steel structures (thin plates) containing elliptical air voids. The procedure for the generation of the dataset is described in our previous work [31]. To give a brief description, PIT data was obtained with MATLAB heat transfer simulations using the parameters of an experimental system where a triggered capacitor discharge through Balcar ASYM 6400 white light lamp delivers a pulse of 6400 J/2ms thermal energy to the materials surface, and a FLIR x8501sc camera (FLIR Systems Inc., Wilsonville, OR, USA) acquires images with 342 pixels spatial sampling resolution at 540 Hz frame rate (180 Hz at full frame rate of 1280 × 1024 pixels). Schematic depiction of the PIT system is shown in Figure 1a. Heat transfer computer simulations generate a database of plate surface temperature transients *T*(*y*,*t*). The plates with stainless steel 316 thermophysical properties have physical dimensions of 5 mm × 5 mm, with a mesh spatial resolution of Δx = Δy = 10 µm, which corresponds to 500 × 500 elements in the computational grid. Elliptical defects are characterized by semi-major and semi-minor axes *R*x and *R*y, and the angular orientation *θ* is measured for counterclockwise rotation from the *x*-axis (the *x*-axis is along the depth of the plate, and the *y*-axis is along the face of the plate)**.** A diagram of an elliptical material defect is shown in Figure 1b. The range of values for both *R*x and *R*y is 10–310 µm, with angular orientations *θ* in the range of 0–45°. The centers of the microscopic elliptical voids were centered along the face of the plate and placed at 1.5 mm depth from the edge of the plate.

The TT algorithm processes the PIT data to obtain reconstructions of the thermal effusivity *e*(*x*,*y*), which is a measure of how a material exchanges heat with its surroundings [31]. Spatial distribution of thermal effusivity allows for the visualization of material defects. The reconstruction algorithm assumes one-dimensional heat diffusion along the *x*-axis (depth of the plate), and obtains *x* or depth dependence of thermal effusivity at each location *y* along the surface of the plate as

(1) $$\;e\left(x,y\right)=x\frac{2Q}{\pi \sqrt{\alpha }}\frac{d}{dt}{\left(\frac{1}{T\left(t\right)}\right)}_{t=x^{2}/\pi \alpha }$$

where *Q* is the instantaneously deposited surface thermal energy density, and *α* is thermal diffusivity. Equation (1) shows that the spatial reconstruction of effusivity *e*(*x*,*y*) is given as a product of depth function *x* and time derivative of the inverse of surface temperature *T*(*t*) evaluated at time *t*. To calculate *e*(*x*,*y*) at a particular value of *x*, we first calculate the corresponding time *t* = *x*^2^/*πα*, and then take the time derivative of the inverse of *T*(*t*) at this time *t*. An example of pseudo color image visualization of simulated thermal effusivity *e*(*x*,*y*) [J∙m^−3/2^∙(mK∙s)^−1/2^] reconstruction for an elliptical defect with *R*_x_ = 60 µm, *R*_y_ = 310 µm, and *θ =* 15^o^ is shown in Figure 1c. The total size of the TT database generated with this procedure is 329 images, where each image has 500 × 342 pixels.

**Figure 2:**
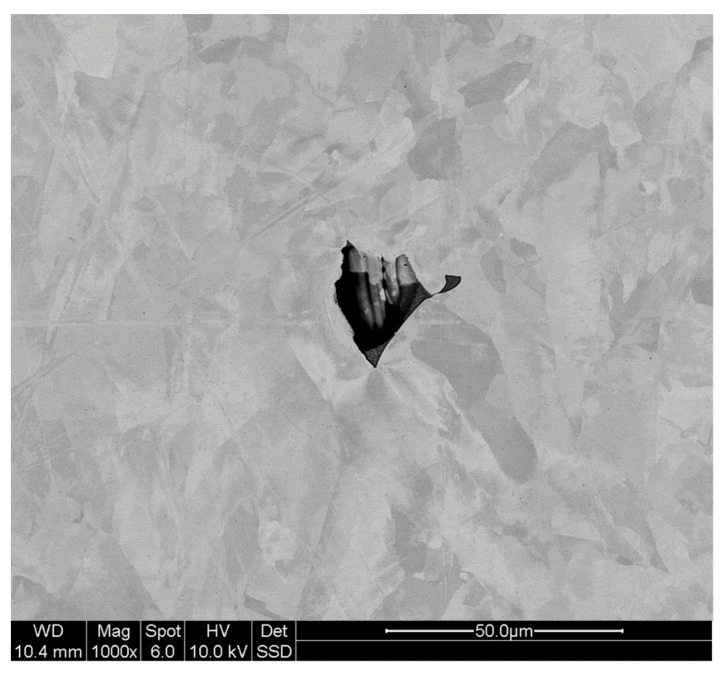
SEM image of material defect in a section of LPBF-printed stainless steel 316.

The dataset for the segmentation task consists of SEM images of sections of LPBF printed stainless steel 316L specimens. Several selected examples from this dataset were used for creating irregular-shaped defect templates in our previous work [31]. The sections contain microscopic irregular-shaped cavities, which are randomly chosen cross-section cuts through irregular-shaped lack-of-fusion pores. Images were acquired with Hitachi S-4700 SEM (Hitachi America Ltd., Santa Clara, CA, USA) with a spatial resolution of 15 nm/pixel. A representative SEM image is shown in Figure 2. The dataset consists of a total of 212 SEM images, but with only 49 images (23% of the total set) containing a material defect. The 163 featureless images were not used in this study. Images were cropped to 224 × 224 pixels size, which is a standard size for deep learning training. The defects were hand labeled using an open source label-studio [50]. The images were converted to a PyTorch tensors format for use in the deep learning library.

### 2.2. Multi-Task Network Architecture

**Figure 3:**
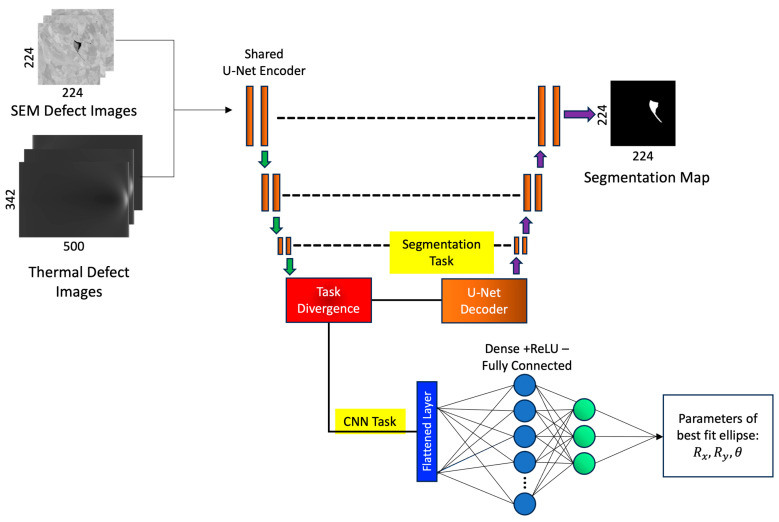
MTL network for simultaneous segmentation of SEM images and parameter classification in TT images. MTL uses a shared U-Net encoder for joint learning.

The MTL network developed for simultaneous regression–segmentation analysis of the TT and SEM images is shown in Figure 3. The TT and SEM images are jointly fed into the network, and the outputs for each task are assessed with the loss functions and evaluation metrics defined in Section 2.6. To implement the MTL, we leverage shared network parameters through a “from-scratch” U-Net encoder. The encoder is trained on images from two different datasets for different tasks, allowing the encoder to learn and extract latent representations shared between the tasks. Subsequently, the MTL network information flow splits into two branches for each specific task, generating a segmentation mask for material defects analysis in the SEM images, and predicting characteristic parameters for the elliptical defects in simulated TT images.

Training of the joint task model occurred over 500 epochs, where both tasks were optimized using Adam optimizer and a learning rate of 10^−5^, a standard rate used with this optimizer [51]. We paired each TT image with one SEM image and used a 65/15/20 training/validation/testing percentage split, with 210 image pairs in training, 53 image pairs in validation, and 66 image pairs in holdout testing. Because the dataset of SEM images contains only 49 entries, to match the size of the database of TT images, we sampled with replacements from the SEM dataset. We also applied data augmentation techniques, such as random rotations of the images.

### 2.3. U-Net Shared Encoder

**Figure 4:**
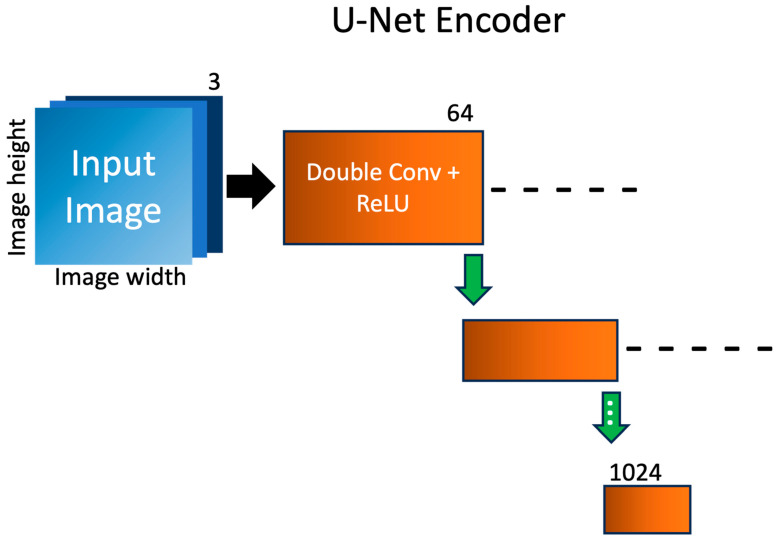
Schematics of the shared U-Net encoder. Each 3-channel image is fed into a double convolutional layer with added non-linearity via ReLU activation to expand the number of channels. Each green arrow represents a max-pooling layer, responsible for reducing the size of the image. Skip connections that connect down-sampled layers to up-sampled layers are shown as dashed lines. For brevity, the double convolutions are combined, and only 2 of the 4 layers are shown.

A shared U-Net encoder is employed in the MTL network, where images from the distinct datasets are jointly fed into the network, as shown in Figure 4. The encoder is designed to progressively down-sample the input images through the repeated application of double 3 × 3 convolutional layers, with each convolutional layer followed by a rectified linear unit (ReLU) activation function. The integration of ReLU activation functions introduces non-linearity to the model, allowing the encoder to learn the complex and abstract patterns present in the data. Inserted between the double convolutional layers are max-pooling layers. Max-pooling serves as a down-sampling mechanism to reduce the spatial dimensions of the feature maps, while retaining the most relevant information. The repeated application of double convolutional layers and max-pooling operations enables the U-Net encoder to progressively reduce the spatial dimension of the feature maps while expanding the number of channels and retaining essential semantic information. One of the key strengths of the U-Net architectures lies in the incorporation of skip connections. Skip connections establish links between the encoder and decoder at various spatial resolutions. Skip connections that connect down-sampled layers to up-sampled layers are shown as dashed lines. As a result, the encoder produces a compact, high-level representation of the input images from both SEM and TT domains, which will be subsequently leveraged by the corresponding task-relevant decoder.

### 2.4. U-Net Segmentation Decoder

**Figure 5:**
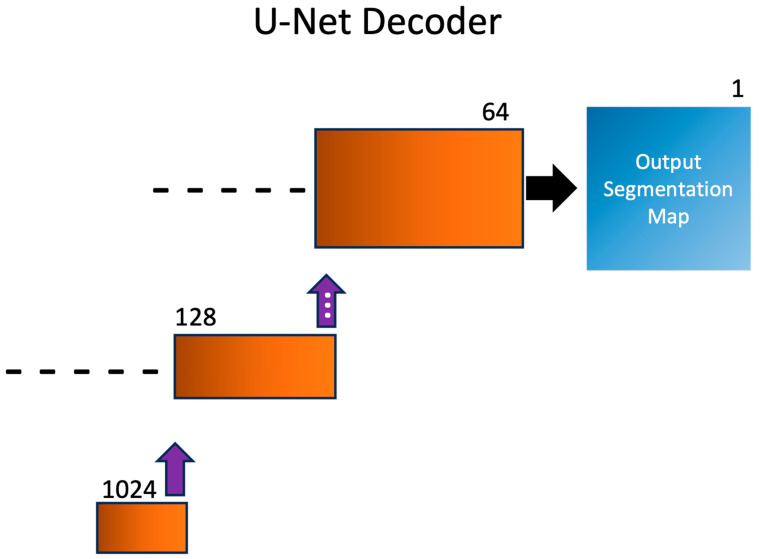
Schematics of the segmentation task U-Net decoder. Spatial information is recovered via several layers of double convolutional layers with ReLU activation, as well as with transpose convolutional layers displayed with purple arrows. A 1-channel segmentation map is output for analysis. For brevity, double convolutions are combined, and only 2 of the 4 up-sampling layers are shown. Skip connections that connect down-sampled layers to up-sampled layers are shown as dashed lines.

After the SEM images are down-sampled by the shared U-Net encoder, the images are sent through the U-Net decoder, shown in Figure 5. The structure of the U-Net decoder utilizes a combination of double convolutional layers and rectified linear unit (ReLU) activation functions, mirroring the structure of the U-Net encoder. In addition to the convolutional layers, the U-Net decoder incorporates transpose convolutional layers (also known as deconvolutional or up-convolutional layers). These transpose convolutional layers serve as an up-sampling mechanism, restoring the resolution lost during the encoders down-sampling phase. This enables the expansion of the channel latent representation and provides a richer spatial context for accurate segmentation. Skip connections that connect down-sampled layers to up-sampled layers are shown as dashed lines. During the down-sampling process, feature maps are copied and concatenated with their mirrored up-sampling feature maps, allowing the decoder access to low- and high-level information simultaneously. This avoids the common issue of vanishing gradients in many machine learning problems. In addition, these features allow the U-Net to perform well in instances with low amounts of training data, as compared to other, more complex algorithms, such as attention transformers. The output of the U-Net decoder is a 1-channel binary segmentation mask where every pixel is classified as either 0 (background), or 1 (feature/defect).

### 2.5. Fully Connected Layer Decoder

**Figure 6:**
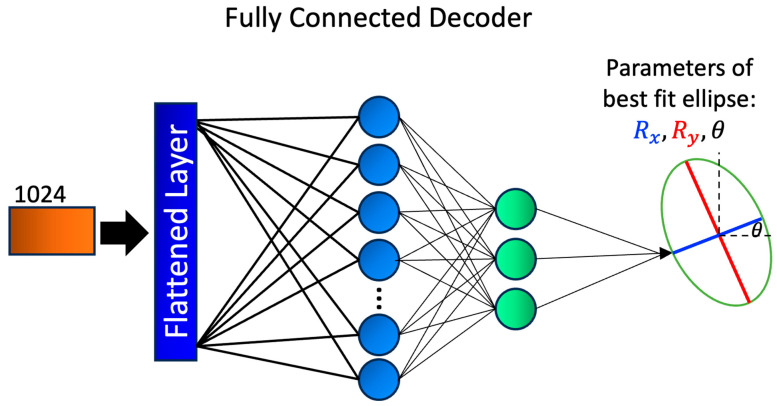
Schematics of the fully connected decoder developed for TT images regression task. The 1024-channel output from the shared encoder is flattened, and then fed through several fully connected linear layers with added exponential linear unit non-linearity. The final layer has a size 3 output, with the corresponding parameters of the best fit ellipse, $R_{x}$, $R_{y}$, and θ.

In designing the elliptical parameter prediction decoder for the TT images, we draw motivation from the previous research where we developed a CNN for the classification of defects in TT images [31]. The CNN takes effusivity reconstruction images as input, and returns characteristic dimensions *R*_x_, *R*_y_, and *θ* of the elliptical defect. To develop the CNN network, in the prior work we used AutoKeras’ image classification module. In this paper, after encoding the TT images with the U-Net encoder, we further process the encoded representations through a series of fully connected layers composed of linear layers and Exponential Linear Unit (ELU) activation functions. ELU activation functions are chosen for their ability to introduce non-linearity, and to prevent vanishing gradient problems. This promotes efficient learning and better representation of complex patterns in the data. The output of the fully connected layers is a compact representation containing three characteristic parameters of the elliptical defect, as shown in Figure 6. To improve generalization and mitigate overfitting, we employ dropout regularization with a probability of 0.2 during training. Dropout randomly deactivates a fraction of neurons during each forward pass, encouraging the network to learn more robust and invariant features. The incorporation of linear layers, ELU activation, and dropout results in a simple yet efficient decoder that can efficiently process the encoded TT images.

### 2.6. Loss Functions and Evaluation Metrics

The prediction of elliptical defect parameters in the TT images is a regressive task. Therefore, we assess the performance of this task with mean squared error (MSE), defined in Equation (2). Here, $Y_{i}$ represents the ground truth value, and ${\hat{Y}}_{i}$ represents the predicted value. The MSE allows us to quantify the extent to which predictions deviate from the true values, providing an overall prediction accuracy. A lower MSE value indicates better performance.

(2) $$MSE=\frac{1}{n}\sum_{i=1}^{n}(Y_{i}-{\hat{Y}}_{i}{)}^{2}$$

Another metric used to assess the performance of the TT images regression task is the area error (AE), defined in Equation (3), as the absolute value in the difference between the areas of the predicted ellipses (*P*), and the actual ellipses (*A*). Here, $\left|P\right|$ represents the absolute value of the area of the predicted eclipse, and $\left|A\right|$ represents the absolute value of the area of the ground truth ellipse. Like MSE, a lower AE value indicates better model performance, suggesting that the predicted ellipse area closely matches the actual ellipse area.

(3) $$AE=\frac{\left|\left|P\right|-\left|A\right|\right|}{\left|A\right|}$$

To quantify the predictive power of our model, we evaluated performance using the Pearson correlation coefficient and the Spearman rank correlation coefficient statistical metrics. The Pearson correlation coefficient, denoted as *Pearson r* defined in Equation (4), measures the linear relationship between predicted values and ground truth values. This coefficient is quantifying the degree of linear dependence between the two variables, where $x_{i}$ is the predicted values for each variable, $\bar{x}$ is the mean of the variables in the sample, $y_{i}$ is the ground truth for each variable in the sample, and $\bar{\;y}\;$ is the mean of the ground truth for each sample. The range of values of *Pearson r* coefficient is from −1 to 1, with values closer to 1 or −1 indicating a stronger positive or negative linear relationship between the variables, respectively.

(4) $$Pearson\;r=\frac{\sum \left(x_{i}-\bar{x}\right)\left(y_{i}-\bar{y}\right)}{\sqrt{\sum (x_{i}-\bar{x}{)}^{2}\sum (y_{i}-\bar{y}{)}^{2}}}$$

The Spearman rank correlation coefficient, denoted as *Spearman r*, assesses the monotonic relationship between predicted and ground truth values. Unlike Pearson *r*, *Spearman r* does not assume linearity in the relationship. Instead, *Spearman r* considers the rank orders of the values, not the raw values of ground truth and prediction. *Spearman r* is defined in Equation (5), where *d* is the difference between the ranks of each sample, and *n* is the number of samples.

(5) $$Spearman\;r=1-\frac{6\sum d_{i}^{2}}{n\left(n^{2}-1\right)}$$

Given the binary nature of the segmentation task, we assess performance using the binary cross entropy (BCE) loss, defined in Equation (6). Here, *y* is the ground truth pixel value $\left(y=1\;or\;y=0\right)$, and *p* is the predicted probability from the model that the pixel belongs to the foreground class (i.e., probability given pixel is a defect, y=1). We utilize BCE due to its heavy penalization on misclassified pixels. This allows the model to focus on the most challenging image regions, placing more emphasis in image regions where defects may occur. Calculating BCE in PyTorch allows us to define the pos_weight parameter. This is a parameter that places more weight on the positive class within each image, which is helpful for the SEM image segmentation task where the distribution of background and foreground pixels is generally unequal.

(6) $$BCE=-\left(ylog\left(p\right)+\left(1-y\right)log\left(1-p\right)\right)\;$$

Finally, a segmentation metric used for better interpretation of each pixel is intersection-over-union (IoU), defined in Equation (7). IoU is interpreted as the ratio of overlap between the model output and the ground truth label over the total surface area of the two parameters.

(7) $$IoU=\frac{\left(prediction\cap truth\right)}{\left(prediction\cup truth\right)}$$

## 3. Multi-Task Learning Image Analysis Results

### 3.1. Classification of the Synthetic TT Images

To evaluate the performance of the joint MTL model, we begin the assessment by individually analyzing each task. First, we assess the performance on the encoder and fully connected layer multitasking model for parameter prediction in synthetic TT images by comparing it to the performance of the single-task model, i.e., the original “from scratch” CNN method developed in prior work [31]. MSE loss values for training, validation, and holdout testing for both multi-task and single-task approaches were calculated for the three elliptical defect parameters (semi-major radius R_x_, semi-minor radius R_y_, and the angle of rotation θ). As an additional metric, we calculated the area error (AE) loss for training, validation, and holdout testing for the single and MTL networks as well. The MSE and AE loss values are listed in Table 1. While the single-task network performs better during training, the stark increase of the testing loss indicates overfitting during training. Despite shuffling the data, applying augmentation techniques, and applying dropout (discussed in Section 2.5), the single task model suffers from overfitting due to lack of variance in the limited size dataset. This further highlights the advantages of multi-task models with disjoint datasets. The steady decrease in the loss values of the multi-task network implies better learning of the underlying trends present in the data. Due to the overfitting, the single-task network has better performance in the AE metric as well. Overall, the multi-task network has better performance when we consider the general trend of predictions.

**Figure 7:**
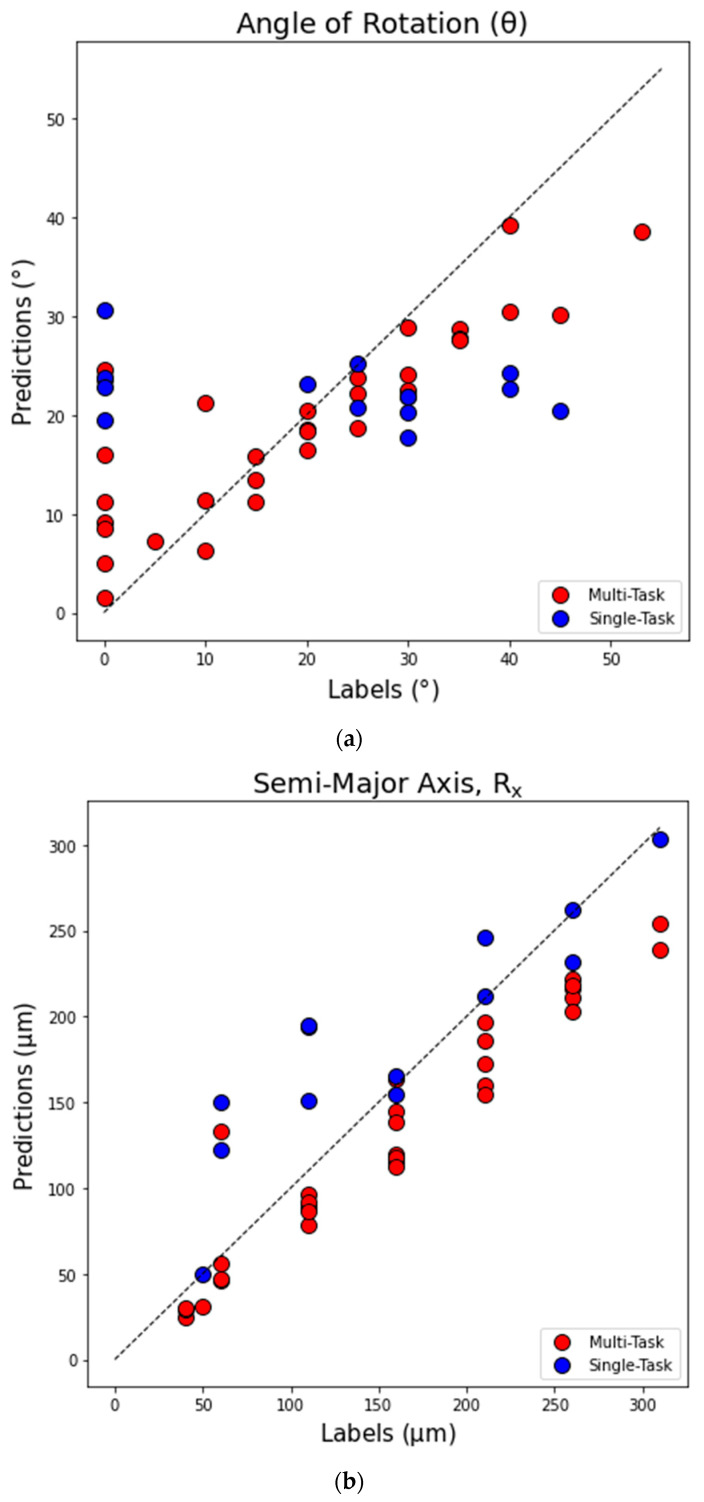

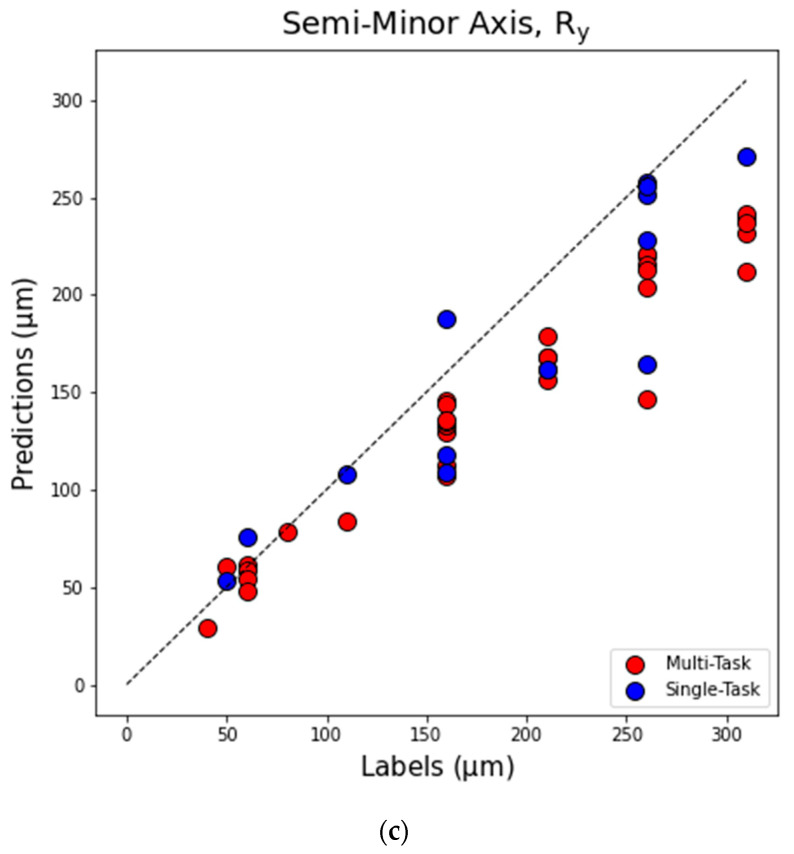
Scatterplot of all holdout testing data predictions vs. labels (true values), where each point represents a single testing instance from the test dataset. Results obtained with multi-task (MTL) and single-task (CNN) networks are plotted with red and blue circles, respectively. (**a**) Angle of rotation *θ* (^o^); (**b**) semi-major axis *R*_x_; (**c**) semi-minor axis *R*_y_ (µm).

For further visualization of the results, we show the scatterplots for the holdout testing data predictions vs. labels (i.e., true values) for the angle of rotation θ (measured in degrees), semi-major axis R_x_, and semi-minor axis R_y_ (measured in microns) in Figure 7a,b,c, respectively. Each point in the scatterplot represents a single testing instance from the test dataset. In all figures, the results obtained with MTL and single-task networks are indicated with red and blue circles, respectively. The values of *Pearson r* and *Spearman r* correlation coefficients for predictions of θ, R_x_, and R_y_ with single-task and multi-task networks for the scatterplots in Figure 7 are listed in Table 2. For reference, a dashed line indicating prediction = labels is drawn in all figures. As can be seen in Figure 7a and the corresponding entries in Table 2, there is a substantial improvement in predicting θ by utilizing the MTL network. The angle θ was poorly predicted by a single-task CNN, while the MTL network produces a much stronger linear correlation between predictions and labels. From Figure 7a, the most challenging cases for prediction for the MTL network are those with θ < 10^o^ and θ > 35^o^. For smaller angles, MTL overpredicts, while for larger angles, MTL underpredicts the results. The same pattern of errors angle predictions is observed for the performance of a single-task network. Results for the predictions of R_x_ and R_y_ with multi-task and single-task networks are shown in scatterplots in Figure 7b,c. Corresponding values of the *Pearson r* and *Spearman r* correlation coefficients in Table 2 indicate that while the single-task network was able to perform reasonably well in predicting the radii, the multi-task network provides a further performance improvement. From Figure 7b,c, performance of the MTL network gradually decreases with increasing length for both R_x_ and R_y_. The error in MTL for both R_x_ and R_y_ is mostly under -prediction of true values, while for single-task CNN networks the error is mostly over-prediction for R_x_ and under-prediction for R_y_.

### 3.2. Segmentation of SEM Images

To evaluate the efficiency of our method in the segmentation of SEM images, we compare the performance of a single-task U-Net model trained “from scratch”, and the MTL network that employs the same U-Net encoder. Values of the binary cross entropy (BCE) loss and mean intersection-over-union (IoU) for training, validation, and testing data for single-task and multi-task networks are summarized in Table 3. While the single-task U-Net model performs reasonably well, we observe an improvement in performance for all datasets when using the MTL model. For example, for the holdout testing data, the single-task U-Net model attained a mean IoU score of 0.81 and a BCE loss value of 0.31. It should be noted that the single-task U-Net model underwent training for 155 epochs before early stopping was implemented, so that the increase in validation IoU score was potentially saturated. For the MTL network processing the holdout data, the IoU score is 0.87 and BCE testing loss is 0.03. These results were obtained after training the MTL for 500 epochs, a comparatively longer training duration for a more complex multi-task model than the single-task model.

**Figure 8:**
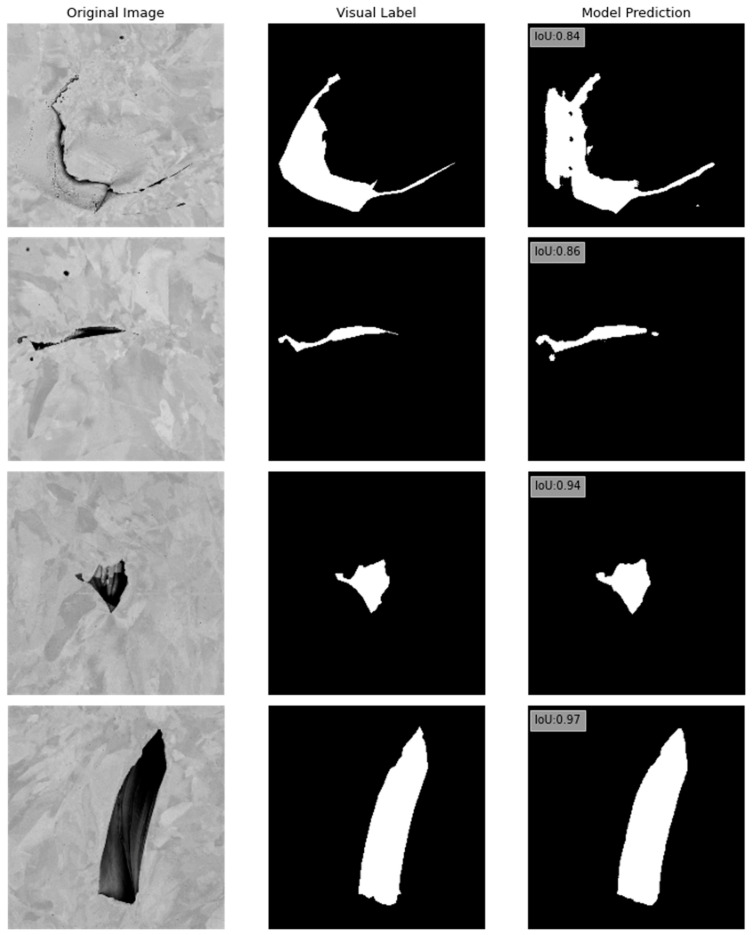
Examples of segmentation map outputs from the MTL model on the holdout testing data. The left column shows the original SEM images. The middle column shows the labeled images. The right column shows the MTL segmentation model predictions, with corresponding testing IoU scores.

Several examples of the segmentation maps are shown in Figure 8. The first column in Figure 8 shows the original SEM images. The second column shows the visual labels. The third column shows the segmentation model predictions, with corresponding testing IoU scores.

Future work on the segmentation of SEM images could involve more sophisticated labeling techniques, such as incorporating self-training semantic segmentation techniques [52]. By leveraging the potential of self-training, the model can iteratively improve its segmentation performance by utilizing its own predictions as pseudo-labels.

## 4. Conclusions

The TT is a promising method for non-destructive imaging of subsurface defects in additively manufactured metals. Because the method is based on heat diffusion, limitations of TT images include blurring, which increases with depth of the defect inside the metal. We have been investigating the enhancement of TT imaging capabilities using machine learning, focusing on CNN-based classification of defects in prior work. In this paper, we have introduced a novel multi-task learning (MTL) approach to simultaneously perform the classification of elliptical defects in synthetic TT images, and the segmentation of SEM images of sections of LPBF-printed SS316 specimens. The results of the MTL model developed in this paper demonstrate improved performance relative to classification and segmentation tasks performed independently. The improved performance indicates the advantages of integrating diverse yet related tasks into a comprehensive framework for the detection of material defects in images. The MTL methodology demonstrated in this work is novel, as MTL models are traditionally designed to process similar datasets, while our methodology applies a unified model to disjoint datasets. While the idea of combining tasks with disjoint datasets is initially counterintuitive, the similarity between the tasks, i.e., that both tasks aim to detect defects within images, becomes apparent at the feature level. The enhanced performance of the MTL model can be attributed to its ability to leverage shared information and feature representations from both tasks, leading to a more comprehensive understanding of defect patterns and, consequently, more accurate classification and segmentation results.

In future work, we plan to extend the MTL model analysis to experimental TT data. Although the study in this paper was based on synthetic TT images, it should be noted that recent research approaches to ML of NDE data involve a combined use of experimental, augmented, and synthetic data. The reason is that, in practice, it is difficult to acquire an experimental NDE database that exhibits sufficient variance to train an ML model that achieves high prediction accuracy. Therefore, the results of the present study provide a valuable benchmark for MTL network performance, which will guide future work on experimental TT data. Another direction for future research could involve analysis on other datasets of images of defects in AM metals obtained with high-resolution methods, such as X-Ray CT, and with different lower-resolution NDE modalities, such as ultrasonic and Eddy current imaging.

## Figures and Tables

**Table 1:** Evaluation of mean square error (MSE) loss and area error (AE) for the MTL network compared to the original “from scratch” CNN method for elliptical defect parameters prediction.

Loss	Single-Task	Multi-Task
Train MSE	12.56	84.23
Validation MSE	21.66	42.06
Test MSE	98.48	38.54
Train AE	0.259	0.09
Validation AE	0.20	0.64
Test AE	0.23	0.47

**Table 2:** Values of *Pearson r* and Spearman ρ correlation coefficients for predictions of elliptical defect angle of rotation *θ*, semi-major axis *R*_x_, and semi-minor axis *R*_y_.

Variable	*θ*		*R*_x_		*R*_y_	
Network	Single-Task	Multi-Task	Single-Task	Multi-Task	Single-Task	Multi-Task
*Pearson r*	−0.34	0.82	0.89	0.96	0.92	0.97
Spearman ρ	−0.28	0.8	0.92	0.96	0.93	0.96

**Table 3:** Binary cross entropy (BCE) loss and mean intersection-over-union (IoU) for a single-task U-Net model, and multi-task learning network that includes U-Net model.

Dataset	Metric	Single-Task	Multi-Task
Training	BCE	0.03	0.01
Validation	BCE	0.03	0.02
Testing	BCE	0.31	0.03
Training	IoU	0.88	0.92
Validation	IoU	0.79	0.92
Testing	IoU	0.81	0.87

## Data Availability

Not applicable.
